# Stakeholders' perspectives on communicating biosecurity to encourage behavior change in farmers

**DOI:** 10.3389/fvets.2025.1562648

**Published:** 2025-03-19

**Authors:** Sebastian Moya, Kate Lamont, Marnie L. Brennan, Giovanna Ciavarino, Maria Costa, Alberto Allepuz, Lena-Mari Tamminen, Carla Correia-Gomes, Helena De Carvalho Ferreira, Mehmet Murat Dogusan, Teresa Imperial, Daniele De Meneghi, Miroslav Kjosevski, Ilias Chantziaras, Alison Burrell

**Affiliations:** ^1^School of Veterinary Medicine and Science, University of Nottingham, Nottingham, United Kingdom; ^2^Centre for Epidemiology and Planetary Health, School of Veterinary Medicine, Scotland's Rural College, Inverness, United Kingdom; ^3^Facultat de Veterinària, Departament de Sanitat i Anatomia Animals, Universitat Autònoma de Barcelona, Cerdanyola del Vallès, Barcelona, Spain; ^4^Department of Clinical Sciences, Swedish University of Agricultural Sciences, Uppsala, Sweden; ^5^Animal Health Ireland, Carrick on Shannon, County Leitrim, Ireland; ^6^Faculty of Health and Life Sciences, Institute of Infection, Veterinary and Ecological Sciences, University of Liverpool, Liverpool, United Kingdom; ^7^Faculty of Veterinary Medicine, Department of Animal Science, University of Burdur Mehmet Akif Ersoy, Burdur, Türkiye; ^8^Dipartimento di Scienze Veterinarie, Università degli Studi di Torino, AgroVet Campus, Grugliasco, Italy; ^9^Faculty of Veterinary Medicine – Skopje, Animal Welfare Center, Department for Animal Hygiene and Environmental Protection, Ss. Cyril and Methodius University in Skopje, North Macedonia; ^10^Faculty of Veterinary Medicine, Veterinary Epidemiology Unit, Ghent University, Merelbeke, Belgium

**Keywords:** communication, behavior change, biosecurity, farmers, focus groups

## Abstract

Effective communication is crucial for strengthening collaboration and ensuring the successful implementation of biosecurity measures against infectious diseases. A collaborative approach, where farmers and veterinarians play a central role in decision-making, may have a greater impact on promoting the implementation of biosecurity practices compared to a top-down approach. The objective of this study was to explore the perspectives of researchers, official services, and industry on the preferred communication methods between farmers and various on-farm stakeholders. Data were collected through four simultaneous focus groups conducted within the framework of the COST Action BETTER project: three involving researchers, and one involving official services and industry people. The data were analyzed using content analysis, which generated three main themes and 13 subthemes: (i) effective methods for communicating biosecurity messages to farmers: direct interaction and practical learning, audio-visual media and support materials, importance of personalization and coordination, and challenges and innovative solutions; (ii) designing an optimal communication system to promote behavioral change in biosecurity: initial strategies for communication: knowledge and trust, integration of technological tools, mandatory programs and coordinated campaigns, continuous training and collaborative learning, and incentives and certifications; and (iii) measuring the success of biosecurity communication programs: evaluation tools and audits, key indicators and benchmarking, measuring attitudes and behavioral changes, and participation and knowledge as additional evaluation metrics. The findings highlight the need for collaborative, personalized, and sustainable approaches to biosecurity communication. This study provides valuable insights to inform the development and implementation of communication programs that remain effective over time.

## 1 Introduction

Communication within animal farming systems, particularly concerning biosecurity, is a complex process involving multiple elements, ranging from message clarity and channel selection to the willingness of participants to engage (1–5). Effective communication not only involves the transmission of information but also depends on factors such as trust among stakeholders, shared perspectives, power dynamics, and accessibility to appropriate communication methods and tools (4–9). Understanding how these factors interact is essential for strengthening collaboration and ensuring the effective implementation of biosecurity measures against infectious diseases, including zoonoses, thereby improving both animal health and public health.

In recent years, various national and international plans have addressed the issue of biosecurity on livestock farms through a top-down approach, whereby stakeholders within the sector often receive mandatory instructions underpinned by regulations, with non-compliance potentially resulting in sanctions (7, 10, 11). However, this strategy has demonstrated limitations, as many stakeholders comply primarily to avoid sanctions or, in some cases, to obtain financial benefits (12). This indicates that a sanction-based approach alone is insufficient to foster genuine and sustained behavioral change in biosecurity practices. Conversely, a collaborative approach may have a greater impact on encouraging effective implementation of biosecurity measures (13).

Two-way communication among various stakeholders, particularly between farmers and veterinarians, has become essential for improving biosecurity practices (3, 4, 10). Both stakeholders play a central role in decision-making on livestock farms, regardless of the type of farming system, and their interactions can significantly influence the implementation of biosecurity measures (14, 15). In light of this, the present study aims to explore the perspectives of researchers, official services, and industry (such as government representatives, official veterinary services, representatives from the industry/private sector (producers), and private veterinarians or consultants) on the communication methods between farmers and various on-farm stakeholders, with the goal of proposing innovative communication strategies to promote behavioral change in biosecurity practices. The findings of this study seek to contribute to the improvement of communication methods and tools used in biosecurity practices.

## 2 Methods

### 2.1 Context

This study builds on a previous survey that explored stakeholders' perspectives on biosecurity communication in livestock farming. The survey was conducted among stakeholders involved in two projects: the COST Action “Biosecurity Enhanced Through Training, Evaluation and Raising Awareness” (BETTER) and the Horizon 2020 “Networking European Poultry Actors for Enhancing the Compliance of Biosecurity Measures for Sustainable Production” (NetPoulSafe).

The survey explored both the communication methods and tools that participants believed were preferred by farmers and those they personally preferred for engaging in biosecurity practices (Supplementary Table S1). It was distributed electronically through the networks of researchers of the COST Action BETTER project. Participants provided informed consent to take part in the study.

Through focus group discussions, this research aimed to explore the initial survey findings in greater depth. Both the survey and the focus groups aimed to explore the perspectives of different stakeholders involved in communicating with farmers, as they were more easily accessible through the COST Action network, a collaborative project between researchers.

### 2.2 Data collection

With some of the same participants, a data exploration was conducted 48 h after the survey through simultaneous focus group discussions (16). The focus groups were held in person during a meeting of the COST Action BETTER project in collaboration with the Horizon 2020 NetPoulSafe project, which took place in Padua, Italy, on 6–7 February 2024. Key survey findings were presented to explore the perspectives of both researchers, as well as official services and industry, prior to the focus group discussions.

Each focus group (facilitated by a leader and supported by at least one note-taker) included up to 20 participants. Groups were formed through the random selection of participants, with the exception of the official services and industry group. The groups engaged in a semi-structured discussion lasting ~1 h, based around a thematic guide with four key questions: (Q1) “You have just seen the results of the survey, what are your thoughts on that? Were you surprised? Do you think differently?” (5 min); (Q2) “What do you think are the most effective methods of communication with farmers?” (10 min); (Q3) “If you were going to design an optimal system for communicating with farmers that leads to a change in behavior regarding biosecurity, how would you do it? What would that look like?” (10 min); and (Q4) “How would you measure the success of this system or program? Do you have examples from existing improvement programs?” (20 min). To facilitate and stimulate discussion, participants were provided with post-it notes and flipcharts to write down their ideas or reflections, which were then individually presented for brainstorming and debate. At the end of the discussions, a volunteer from each group summarized their group's results during a presentation (without discussion) in a plenary session to the other groups. During the plenary presentations, volunteers were allocated a maximum time of ~5 min, with optional use of visual aids. These presentations were recorded and subsequently transcribed for analysis.

### 2.3 Data analysis

Data from the post-it notes, flipcharts, discussion group notes, and plenary presentations, were transcribed and organized by focus group. These documents were analyzed using content analysis to organize and extract significant patterns from the data collected (17). The data were manually coded and categorized in Microsoft Word into themes and subthemes, while maintaining the identification of each focus group. In fact, in the results, the identification has been kept in parentheses to indicate the source of each finding (e.g., FG1 referring to the first focus group). Although there were no differences in the composition of the researcher groups, they were separated in the results, mainly because the sample collection differed between groups. The themes were derived deductively from the thematic guide, while the subthemes, which were recurrent, were identified inductively from different sections of the text (18). The analysis enabled the interpretation of intra- and inter-group trends and relationships, highlighting shared or divergent perspectives between researchers, official services and industry.

## 3 Results

### 3.1 Survey

The prior survey was completed by 51 respondents, with 48% identifying as female and 52% as male. Respondents ranged in age from 25 to 67 years, with an average age of 42. The majority (40.78%) were researchers (mainly involved in projects related to biosecurity and therefore linked to animal health issues), followed by government representatives (4.8%), individuals in “other roles” (4.8%), official veterinary services (2.3%), and one (person) private veterinarian or consultant. Participants represented a wide geographic scope, spanning 22 countries.

In terms of interaction with farmers, 62% (31) of respondents reported engaging with farmers multiple times per year, 20% (10) interacted less frequently, and 18% (9) indicated they rarely interacted with farmers. More frequent interactions with veterinarians were reported: 78% (39) of respondents estimated that, based on their experience, farmers interacted with veterinarians several times a year, 12% (6) believed that these interactions were less frequent, and 10% (5) believed that they were rare.

The results indicated a certain degree of agreement between the communication methods perceived to be preferred by farmers and those that the respondents actually employed when engaging with them (Table 1). The respondents reported that on-site farm visits and face-to-face group meetings were the primary communication methods they used, which also aligned with the methods perceived as mostly preferred by farmers, although the frequency of perceived preference was slightly higher than actual use. Furthermore, the responses to webinars, online seminars, individual online meetings, and online resources (e.g., websites) showed correspondence between their limited use and the perceived farmers' preferences. However, discrepancies emerged in specific methods, particularly telephone conversations, which were used more frequently than they were perceived to be preferred by farmers. Printed materials, such as leaflets and pamphlets, also showed moderate differences, whereas written correspondence remained consistently low in both use and perceived preference.

**Table 1:** Results from two sets of questions on methods of communicating with farmers about biosecurity.

**Method**	**What methods do you use/would you use to communicate with farmers about biosecurity?**		**What communication methods do you think farmers prefer?**	
	***n***	**Total**	***n***	**Total**
I do not engage	9	51	4	51
Printed leaflets or pamphlets	14	51	7	51
Educational videos	20	51	15	51
Written correspondence (letters)	7	51	1	51
Telephone conversations	22	51	12	51
Individual online meetings	10	51	7	51
Webinars or online seminars	14	51	10	51
On-site farm visits	38	51	43	51
Face-to-face group meetings	30	51	35	51
Online resources and websites	13	51	9	51
Other	1	51	1	51
Total responses	178		144	

### 3.2 Focus groups

**Figure 1:**
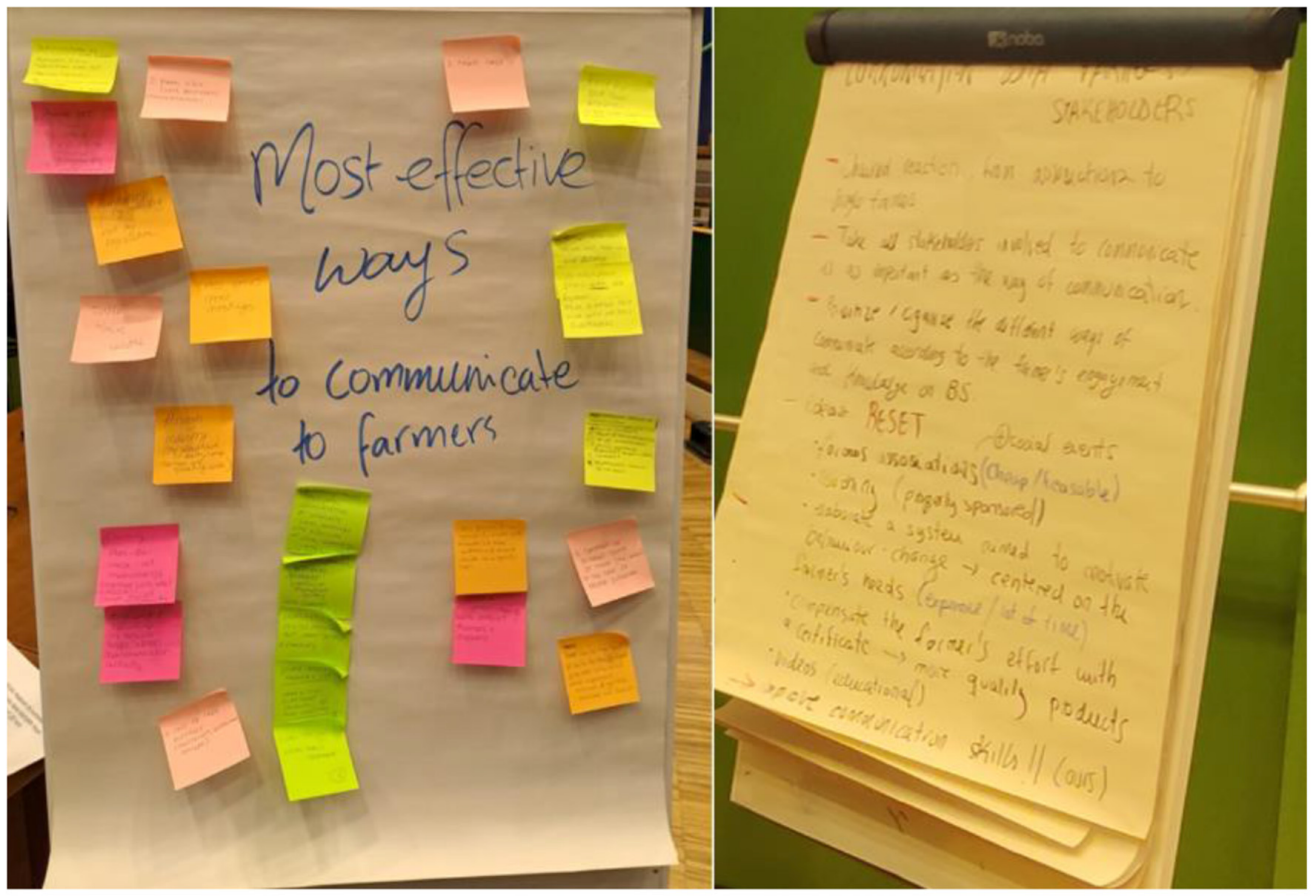
Post-it notes and flipcharts created by focus groups 1 and 4.

A total of 54 participants, four facilitators, and seven note-takers took part in four focus group discussions. Three groups were composed of researchers, and the other one was composed of official services and industry people. Details of each group and the materials used for analysis are provided in Table 2. The selected records from the post-it notes and flipcharts are presented in Figure 1. In total, 3 themes and 13 subthemes were developed (Table 3).

**Table 2:** Details of focus groups and materials used for analysis.

**Focus group (FG)**	**Group member composition in the focus group discussions**			**Materials utilized during the focus group discussions**			
	**Facilitators and note-takers**	**Number of participants**	**Gender distribution of participants**	**Post-it notes**	**Flipcharts**	**Discussion group notes**	**Plenary presentations**
FG1: Official services and industry	AB^*^, CC-G	9	6F/3M	Yes (Q2); No (Q1, Q3-4)	Yes (Q3-4); No (Q1-2)	Yes	Yes
FG2: Researchers	DDM^*^, HCF, IC, NC	12	6F/6M	Yes (Q1-4)	No (Q1-4)	Yes	Yes
FG3: Researchers	AA^*^, MLB, SM	16	5F/11M	Yes (Q2-4); No (Q1)	No (Q1-4)	Yes	Yes
FG4: Researchers	MK^*^, L-MT	17	6F/11M	No (Q1-4)^**^	Yes (Q2); No (Q1, Q3-4)	Yes	Yes

F, Female; M, Male; Q, Questions; ^*^Facilitators. ^**^At least for one question, they were used but were not found for analysis.

**Table 3:** Themes and subthemes developed from the content analysis.

Effective methods for communicating biosecurity messages to
farmers
- Direct interaction and practical learning
- Audio-visual media and support materials
- Importance of personalization and coordination
- Challenges and innovative solutions
**Designing an optimal communication system to promote**
**behavioral change in biosecurity**
- Initial strategies for communication: knowledge and trust
- Integration of technological tools
- Mandatory programs and coordinated campaigns
- Continuous training and collaborative learning
- Incentives and certifications
**Measuring the success of biosecurity communication programs**
- Evaluation tools and audits
- Key indicators and benchmarking
- Measuring attitudes and behavioral changes
- Participation and knowledge as additional evaluation metrics

#### 3.2.1 Effective methods for communicating biosecurity messages to farmers

For effective communication about biosecurity with farmers, participants recommended a multidimensional approach that considered their specific needs, interests, and contexts. Researchers, official services, and industry reached agreement on several aspects, although they put forward nuanced proposals, including: (i) direct interaction and practical learning, (ii) audio-visual media and support materials, (iii) the importance of personalization and coordination, and (iv) challenges and innovative solutions.

##### 3.2.1.1 Direct interaction and practical learning

Researchers (FG2, FG3, and FG4), official services, and industry (FG1) emphasized face-to-face contact, especially through farm visits, as one of the most effective communication methods. These visits were thought to help establish trust (FG1 and FG2). Researchers (FG2) highlighted that addressing on-site issues and finding solutions with farmers strengthens the implementation of biosecurity measures, while official services and industry (FG1) underlined the role of veterinarians and advisors as key stakeholders due to their trusted relationships with farmers. However, farm visits demand resources, in particular time, which may hamper the effectiveness of this method of communication in practice (FG4).

Additionally, researchers (FG2) pointed to practical learning as a key tool, proposing activities such as exchange visits, hands-on demonstrations (“learning by doing”), and educational games (e.g., case studies and exercises) designed to simulate disease spread. These activities were felt to not only promote knowledge acquisition but also foster mutual understanding among farmers.

##### 3.2.1.2 Audio-visual media and support materials

All stakeholders highlighted the usefulness of audio-visual materials, such as videos, especially those highlighting success stories or providing clear instructions on biosecurity measures. Researchers (FG4) suggested short videos to capture farmers' attention in an accessible and engaging format, while official services and industry (FG1) mentioned using tools like social media and farmer “influencers” to counteract misinformation.

Researchers (FG3) stressed that the content of any material must be accompanied by clear instructions or step-by-step protocols aligned with global biosecurity standards. However, they warned that methods relying solely on websites or written texts might be insufficient for achieving communication goals.

##### 3.2.1.3 Importance of personalization and coordination

Message personalization was a recurring theme. Researchers (FG3 and FG4), official services, and industry (FG1) noted that a communication plan tailored to farmers' characteristics and needs is crucial. The groups emphasized the importance of messengers learning to listen, adapting their approach accordingly, and remaining flexible and adaptable. Researchers (FG3) highlighted the importance of reflecting on questions such as, “Why would farmers do this or why wouldn't they?” to design more effective strategies.

Additionally, researchers (FG4) highlighted that communication should be a coordinated effort among veterinarians and other key stakeholders, potentially using frameworks like the RESET (Rules, Education, Social pressure, Economics, and Tools) model to encourage behavioral change (19). They cautioned that inconsistent messages among stakeholders could reduce the effectiveness of biosecurity strategies.

##### 3.2.1.4 Challenges and innovative solutions

A key challenge identified by researchers (FG4) was how to engage farmers during routine circumstances and in the absence of critical situations, such as outbreaks or specific problems. Strategies proposed included creating funded model farms, to serve as examples, and organizing round tables or group discussions to encourage technical exchange and collaborative learning (FG3 and FG4).

Alternative methods such as phone or video calls were also mentioned, which, while considered to be less effective than in-person visits, were reported to help overcome logistical barriers to farm-to-farm visits (FG3 and FG4). However, official services and industry (FG1) stressed that any strategy must be backed by cross-sector learning efforts, involving all stakeholders.

#### 3.2.2 Designing an optimal communication system to promote behavioral change in biosecurity

Discussions on how to design an optimal system for communicating biosecurity messages and driving behavioral change revealed various complementary approaches from researchers, official services, and industry. These approaches included: (i) initial strategies for communication: knowledge and trust, (ii) integration of technological tools, (iii) mandatory programs and coordinated campaigns, (iv) continuous training and collaborative learning, and (v) incentives and certifications.

##### 3.2.2.1 Initial strategies for communication: knowledge and trust

There was consensus on the need to begin by improving farmers' knowledge of biosecurity. Researchers (FG2) suggested that the first step is to communicate risks and benefits of biosecurity practices more effectively. Proposed approaches ranged from soft strategies based on dialogue and persuasion to more robust ones, including inspections followed by tailored support (FG3). Moreover, researchers emphasized that trust is crucial for farmers to adopt new measures (FG3).

##### 3.2.2.2 Integration of technological tools

The use of technology was widely discussed. Official services and industry (FG1) proposed using artificial intelligence (AI) to allow farmers to input their data and receive personalized biosecurity recommendations, stressing the need to combine this technology with human advice. Researchers (FG4) suggested developing apps with gamified elements, such as biosecurity games, to facilitate practical learning.

##### 3.2.2.3 Mandatory programs and coordinated campaigns

Official services and industry (FG1) discussed the role of mandatory programs, in particular for farmers with substandard biosecurity practices. While recognizing their potential, they warned that they might cause resistance if not handled appropriately. They proposed industry-led programs instead of relying exclusively on legislation, as they were believed this could ease acceptance.

Researchers (FG3) suggested the benefit of coordinated campaigns that deliver a unified message through various media, accompanied by tailored materials for veterinarians and farmers. They suggested an approach of adapting successful public health campaign strategies to animal health biosecurity.

##### 3.2.2.4 Continuous training and collaborative learning

All stakeholders agreed on the importance of continuous training as a cornerstone for behavioral change. Researchers (FG4), official services, and industry (FG1) highlighted the need for group workshops and discussions focused on solving specific problems, promoting knowledge exchange among farmers, such as creating farmer clubs (FG4).

Additionally, researchers (FG3 and FG4) underlined the value of in-person and virtual courses led by recognized farmers, complemented by tools like podcasts, social seminars, and groups on platforms such as WhatsApp or Facebook (FG4).

##### 3.2.2.5 Incentives and certifications

To encourage the adoption of better practices, all stakeholders discussed the importance of incentives. Official services and industry (FG1) suggested adding value to products through premium pricing or discounts for those implementing good practices. Researchers (FG4) proposed private certification systems, e.g., badges.

#### 3.2.3 Measuring the success of biosecurity communication programs

Researchers, official services, and industry proposed various methods and concrete ideas: (i) evaluation tools and audits, (ii) key indicators and benchmarking, (iii) measuring attitudes and behavioral changes, and (iv) participation and knowledge as additional evaluation metrics.

##### 3.2.3.1 Evaluation tools and audits

Evaluation tools were widely discussed. Researchers (FG2, FG3, and FG4) recommended using systems to measure biosecurity progress through regular audits (FG2 and FG3). However, they warned that audits should be conducted in farmer-friendly environments to facilitate positive engagement (FG2). Researchers (FG2 and FG4), official services, and industry (FG1) suggested self-assessments for farmers to monitor their progress. All stakeholders proposed a step-by-step approach considering each farm's initial situation (FG1 and FG4) and employing the KISS (Keep It Simple, Stupid) principle (FG1) (20, 21).

##### 3.2.3.2 Key indicators and benchmarking

There was consensus on using tangible indicators to evaluate biosecurity progress. Official services and industry (FG1) suggested health parameters, such as reduced antimicrobial use and decreased infectious diseases, and productive parameters. They highlighted benchmarking as a useful tool for comparing progress among farmers and over time. Researchers (FG2 and FG3) expanded this view to include indicators like animal welfare and environmental metrics, such as water quality in aquaculture. They also noted the absence of outbreaks over extended periods as a sign of biosecurity success (FG3).

##### 3.2.3.3 Measuring attitudes and behavioral changes

Social and psychological factors were considered essential for assessing program impact. Official services and industry (FG1) emphasized the importance of delivering repeated messages in various ways. They cited examples such as the milking gloves campaign, which primarily aimed to communicate that farmers must wear gloves as part of behavioral change (22).

Researchers (FG4) proposed the measurement of change in farmers' attitudes toward biosecurity. In addition, a mechanism to capture the perceptions of farmers about the impact of the measures they had implemented.

##### 3.2.3.4 Participation and knowledge as additional evaluation metrics

Researchers (FG4) highlighted the number of farmers participating in biosecurity-related activities as a key indicator of success. They also suggested measuring farmers' level of biosecurity knowledge (i.e., benchmarking) to assess their understanding of key concepts.

## 4 Discussion

The findings of this study reveal stakeholders' perspectives on biosecurity communication to encourage changes in farmers' behavior. In relation to the focus groups, within the section ‘Effective methods for communicating biosecurity messages to farmers', direct interaction between stakeholders was emphasized by researchers, official services, and industry. It has been previously identified as crucial for the implementation of biosecurity measures within organizational contexts (23), such as on livestock farms. However, depending on the approaches adopted by those involved in the interaction, certain issues may arise. Farm visits conducted without a collaborative focus can lead to rejection from farmers due to the perception of inspection rather than support, as is often the case with government agent visits (10). This study also underscores the importance of ensuring that such interactions promote the co-creation of solutions tailored to the specific context of each farm—an approach that has proven effective in advancing agro-ecological knowledge (24, 25), as well as innovation in farmer field schools (26) and the greenhouse industry (27), among others.

On the other hand, there was consensus on the importance of tailoring biosecurity messages to the farming system and its context. This concern reflects a trend toward the failure of generalized messaging, which often overlooks the specific characteristics and resources of individual farms (28). Furthermore, non-contextualized messages can lead to mistrust and resistance among stakeholders (29), adding an additional barrier to the implementation of biosecurity measures. Designing strategies that incorporate these specificities not only increases the likelihood of implementing biosecurity measures but also ensures that such measures are sustainable in the long term. Selecting the appropriate communication channels for each context is also crucial, considering factors such as farmer demographics, access to technology, and preferred learning styles (30).

Within the section “Designing an optimal communication system to promote behavioral change in biosecurity”, one of the key elements identified was the need to improve biosecurity knowledge among farmers. However, there are divergences in the literature regarding the extent of this knowledge. Some studies argue that farmers possess a basic level of understanding that enables appropriate comprehension of biosecurity practices (3), while others suggest significant gaps in their knowledge (31). This disparity could be attributed to differences in farming systems and geographical locations, but also to the communication strategies themselves, such as the content delivered. Therefore, further research is needed to accurately identify which specific areas of knowledge require strengthening, enabling the design of more effective messages.

On the other hand, there was consensus among researchers, official services, and industry that group discussions with farmers, as a form of training designed to address specific biosecurity issues, could be instrumental in promoting behavioral change. While such participatory training approaches can positively impact biosecurity practices (32), the existing literature lacks studies that clearly define the ideal methodology for this specific type of training. Furthermore, existing training programs, such as those promoted by private projects like FarmIQ's “Farm Biosecurity in Practice” (33) are often designed without the active participation of stakeholders, which may reduce their practical applicability in the field. Therefore, adopting a participatory approach to biosecurity training adds significant value. However, as this approach requires time and a mutual understanding of the needs of all stakeholders, it is essential to combine efforts across all parties, particularly during the design phase, to ensure its efficiency, effectiveness, and sustainability.

Within the section, “Measuring the success of biosecurity communication programs”, one aspect discussed was the self-assessment process conducted by farmers. Self-assessment was proposed as a key tool that goes beyond the use of checklists commonly applied in evaluations, which are not always optimal (34). While this tool could include a farm-specific, personalized approach, it could also incorporate a reflective component. This would enable farmers, alongside other stakeholders, to critically identify areas for improvement, thereby increasing their commitment to biosecurity, similar to findings in other fields such as language studies (35).

This study highlighted a difference in the approach adopted by researchers, official services, and industry. While some official services and industry seemed to position themselves as just another stakeholder in the system, others seemed to position themselves differently. Some researchers, particularly veterinarians, often adopt a paternalistic approach, as highlighted in the literature (15, 36), determining what should be done and how it should be done, without actively engaging or collaboratively seeking solutions. This approach has been criticized in various studies, both in animal and human health contexts (36, 37). Recognizing researchers as just another stakeholder within the system could encourage greater integration and collaboration. This approach would enable researchers to work alongside farm stakeholders (official services and industry). By doing so, some researchers, particularly veterinarians, could gradually move away from adopting a paternalistic stance, as is already practiced by some veterinarians in small animal practice (38–40).

Among the limitations of this study, the small number of official services and industry participants in the focus group discussions can be highlighted. It is crucial to involve and correctly identify stakeholders (via e.g., stakeholder mapping (41))—in this case, official services and industry—who are directly engaged with the subject of study as participants, to achieve a greater impact on the implementation of biosecurity measures through effective interventions (42, 43). It is essential that stakeholders from livestock farms themselves propose strategies to improve communication, adopting a non-hierarchical approach in which researchers primarily play a facilitating role. Furthermore, researchers should also be considered as subjects of study, as seen in certain initiatives in animal health, such as work package one of the BIOSECURE project (44), or previous efforts in human health (45).

While this study examined the communication methods and tools used to promote biosecurity, it did not explicitly analyze the nuances of communication style, the specific vocabulary associated with collaborative approaches nor the impact of language barriers in effective communication. In terms of communication style, biosecurity communication strategies could be strengthened by understanding how language choice and tone influence engagement. In terms of vocabulary, examining how it aligns with collaborative approaches -such as inclusive and participatory language- could improve the effectiveness of biosecurity messaging.

Inclusive language should also consider the written and spoken language skills of other stakeholders involved in the implementation of biosecurity measures, in particular farm workers. Addressing language barriers is essential to ensure effective implementation of biosecurity measures, primarily through adequate training of these stakeholders (46–48). Therefore, inclusivity should be a key element of any communication plan involving all stakeholders.

Future research should explore these aspects in more depth to provide practical insights for stakeholders involved in biosecurity communication.

This study looked at accessibility to appropriate communication methods and tools. However, future research could also explore other aspects of communication not covered in this study, such as trust between stakeholders, shared perspectives and power dynamics, from a collaborative perspective involving all stakeholders, including researchers.

In conclusion, this study offers an initial exploration of the perspectives of researchers, official services, and industry on communication strategies for promoting behavioral change among farmers in relation to biosecurity. It highlights the need for collaborative, personalized, and sustainable approaches to biosecurity communication. However, it does not delve deeply into the various aspects of communication which future studies are recommended to address. This would facilitate the design of communication programs that remain effective over time. While researchers can offer valuable insights and serve as a reference point, these strategies should ultimately be shaped by the perspectives of key stakeholders in livestock farming, including official services, industry, and, crucially, farmers themselves. In fact, this study explored how, from their perspective, biosecurity communication with farmers should be approached by those responsible for education and message dissemination. However, future research should present these ideas directly to farmers, alongside evaluating successful training programs to identify the key elements of effective communication. Additionally, incorporating a participatory action research approach could improve biosecurity communication strategies by promoting co-creation, ensuring that messages are contextually relevant, engagement is more meaningful, and solutions are co-designed to improve uptake and long-term sustainability (25, 49). To build on this, it will be essential to triangulate these perspectives by directly engaging farmers to compare their views on communication with those expressed by researchers and other stakeholders. This would allow for a more comprehensive understanding of how communication strategies can be tailored to meet the needs and expectations of all parties involved.

## Data Availability

The raw data supporting the conclusions of this article will be made available by the authors, without undue reservation.
