# Supplementary material for: Stakeholders' perspectives on communicating biosecurity to encourage behavior change in farmers
Source: Front Vet Sci. 2025 Mar 19;12:1562648. doi: 10.3389/fvets.2025.1562648 (PMC11964111; doi:10.3389/fvets.2025.1562648)
Supplement: Supplementary file 1 [file Data_Sheet_1.pdf]

Supplementary material

Table 1: Survey questions
1. Demographics and profiling
1.1. Age (free numerical answer)
1.2. Gender<br>• Male,<br>• Female,<br>• Non-binary,<br>• Other,<br>• Prefer not to answer
1.3. Country (where you live in)
1.4. Do you interact with farmers often? (often = multiple times per year)<br>• Yes,<br>• No,<br>• Less often than that
1.5. Do you interact with vets often? (often = multiple times per year)<br>• Yes,<br>• No,<br>• Less often than that
1.6. Which stakeholder group do you belong to? (4-6 categories, please choose one)<br>• Researchers/Academy,<br>• Government representatives,<br>• Official veterinary services,<br>• Representatives of the industry / private sector (producers),<br>• Private vets and consultants
2. Key questions
2.1. Q1 – What methods do you use/would you use to communicate with farmers about biosecurity? (tick all that apply)<br>• I do not engage in communication with farmers about biosecurity<br>• Printed leaflets or pamphlets<br>• Educational videos<br>• Written correspondence (letters)<br>• Telephone conversations<br>• Individual online meetings (1-1)<br>• Webinars or online seminars<br>• On-site farm visits<br>• Face-to-face group meetings<br>• Online resources and websites<br>• Other
2.2. Q2 – What methods of communication do think FARMERS would prefer? (tick all that apply)<br>• I do not engage in communication with farmers about biosecurity<br>• Printed leaflets or pamphlets<br>• Educational videos<br>• Written correspondence (letters)<br>• Telephone conversations<br>• Individual online meetings (1-1)<br>• Webinars or online seminars<br>• On-site farm visits<br>• Face-to-face group meetings<br>• Online resources and websites<br>• Other
